# A Comparative Study of the Communication Profile of Typically Developing Children and Children with Receptive-Expressive Language Disorders: A Parental Perceptive

**DOI:** 10.2174/1745017902117010177

**Published:** 2021-12-01

**Authors:** Aiswarya L. Varghese, Chinnu Thomas, Megha Mohan, Sudhin Karuppali

**Affiliations:** 1 Department of Audiology and Speech Language Pathology, Kasturba Medical College, Mangalore, Manipal Academy of Higher Education, Manipal, India

**Keywords:** Children, Concern, Disorder, Expressive, Parent, Receptive, Typically

## Abstract

**Background:**

Parental concerns pertaining to communication abilities are essential as it does aid in the identification of the children at risk of physical and mental health problems.

**Objectives:**

The current study followed a cross sectional study design. The study focussed on developing a questionnaire targeting the parental concerns in Typically developing (TD) children and children with Receptive-Expressive Language Disorders (CWRELD) between 3.7 and 6.6 years of age; to administer the developed questionnaire on parents of TD children and CWRELD; and to analyse and compare the concerns faced by parents of TD children and CWRELD across 3.7 and 6.6 years of age.

**Methods:**

Fifty-one parents of TD children and 51 parents of CWRELD participated in the study. The study was carried out in three phases- Phase I included the development and validation of questionnaire; Phase II included data collection using the developed questionnaire; and Phase III included performing statistical analysis. Descriptive statistics was done to determine the mean and standard deviation (SD) for both the TD and CWRELD groups.

**Results:**

The results revealed that the concerns exhibited by parents of CWRELD were significantly higher than that of parents of TD children. Chi square results indicated statistically significant findings across all the domains between TD children and CWRELD (p<0.05).

**Conclusion:**

The developed questionnaire can be used in clinical settings to help track parental concerns which may aid in the early identification of children at risk of various communication disorders. Additionally, this questionnaire may be considered for monitoring parental concerns throughout the course of the intervention program.

## 1 INTRODUCTION

Communication is the basis of understanding and expressing self, others and surroundings. Parents, who share an intricate relationship with their children, do play a vital role in their children’s communication development. It is, therefore quite natural for parents to have concerns pertaining to their communication, with these concerns playing a key role in the lives of the children [1]. These concerns regarding their child’s development are the worries which may be influenced by the parent’s personality characteristics [2], parental separation anxiety [3, 4], cognition [4], and/or social support. The concerns encountered by parents need not only be associated with children with language disabilities but also children who are typically developing. Parents of the typically developing children do face concerns regarding their child’s education, health, relationship between peers, communication, behaviour, and upbringing. In contrast, parents of children with disabilities are exposed to extended challenges, requiring lifelong care, being subjected to discrimination, and having an increased risk for socioeconomic difficulties [5]. Parental concerns have been observed in children with autism spectrum disorders (ASD) [6], hydrocephalus [7], intellectual disabilities [8], and cerebral palsy [9]. However, the degree and type of concerns may vary depending on how they react to the situation. Variations in the extent and amount of expression of parental concerns can reflect the socio-economic status, gender of the parent and child, and age and cultural background [10]. It is thus useful for researchers, service providers, and clinicians to quantify the level of parental concerns, and estimate the risk of children having developmental problems in the general population, and identify vulnerable subpopulations [11]. The communication skills portrayed by children pose a serious concern to parents. With the communication difficulties encountered in children not being evident in the preschool period [12], concerns may eventually manifest in the domains of receptive and expressive language skills, socio emotional skills, and specifically pragmatic skills. The parental concerns, priorities, needs and strengths need to be taken into account along with the needs of children with disabilities.

The significance of parental concerns in the detection of children with mental health problems has been extensively studied. In a study done in Australia, the signs used to identify impairments in speech and language did include parent-rated concerns of expressive and receptive language, along with the usage of speech-language pathology services, and reduced scores in the receptive vocabulary test [13]. Studies have noted that families with children having developmental disabilities commonly experience higher stress levels [14-17]. Increased stress and depressive symptoms are noted to be associated with parents of children with ASD [6, 18]. When compared to parents of typically developing children or children with other disabilities, studies have found parents of young children with ASD to experience higher stress levels. Parenting stress may increase as the child develops, with the communication gap between the parents and their children becoming eventually more pronounced [19].

The lack of knowledge of parental concerns creates a barrier for assessment and management. Parents being the primary communication partner, it becomes essential for speech language therapists to determine the type of concerns parents go through when it comes to communication skills. Therefore, parents become a crucial and integral part of the rehabilitation team. Sometimes an excessive or lack of concern may have an adverse effect on the child’s well-being. Parental concerns do aid in the identification of the children at risk of physical and mental health problems. Thus, there is a need to address parental concerns, which is one of the predictive factors of children’s problems by health professionals. In order to understand the type of concerns faced by the parents of children with communication disabilities, it also becomes essential to understand the concerns faced by parents of typically developing children. This area of study which is least explored can be carried out in the form of questionnaires which have been vastly used in the western population. However, adapting a culturally different questionnaire towards an Indian society may have its own ramifications. Therefore, the objectives of the current study were: (1) to develop a questionnaire targeting the parental concerns in typically developing (TD) children and children with Receptive - Expressive Language Disorders (CWRELD) between 3.7 and 6.6 years of age; (2) to administer the developed questionnaire on parents of TD children and CWRELD; and (3) to analyse and compare the concerns faced by parents of TD children and CWRELD.

## 2 MATERIALS AND METHODS

The participants included in this study were TD children and CWRELD between 3.7 and 6.6 years of age. A cross-sectional design along with a convenience sampling procedure was employed to compare the parental concerns across the groups. Ethical approval was received from the Institutional Ethical Board before the commencement of the study. The procedures followed in the current study were in accordance with the Helsinki declaration of 1975, as revised in 1983.

### 2.1 Participants

Fifty-one parents of TD children and 51 parents of CWRELD between 3.7 and 6.6 years of age participated in this study. The participants were divided into 6 groups with equal age (6 month) intervals. The details of the participants under each age group have been mentioned in Table **1**.

The participants were recruited based on the following selection criteria. The inclusion criteria included parents of TD children between 3.7 and 6.6 years of age under their respective age groups who have clinically normal speech and language skills. Parents of CWRELD between 3.7 and 6.6 years of age whose diagnosis was ascertained using the Assessment of Language Development [20] were included in the study. The exclusion criteria included parents with a history/diagnosis of psychological or psychiatric behaviours as well as the parents of TD children who have a history/complaint of any speech, cognitive, hearing problems, or any other language related disorder other than RELD.

### 2.2 Procedure

The parents were explained about the purpose of the study, and informed consent was obtained prior to the initiation of the study. The study was conducted within the hospital premises in the city of Mangaluru, between 1^st^ September 2019 and 30^th^ January 2020. The study was carried out in three phases - Phase I: Development of questionnaire; Phase II: Data collection; and Phase III: Statistical analysis. In phase 1, a questionnaire was conceptualized with the intention of addressing the concerns of parents pertaining to the communication abilities of their children. The questionnaire was developed based on the literature review and usage of the existing standardized tools that targeted age specific communication skills of children with communication disabilities. The questionnaire is comprised of 5 domains - (1) Concerns related to the understanding and use of phonology (PH); (2) Concerns related to the understanding and use of morpho-syntax (MS); (3) Concerns related to the understanding and use of semantics (S); (4) Concerns related to the understanding and use of pragmatics (P); and (5) concerns that addressed general (GC) aspects. Each domain consisted of a series of items, which were categorized specific to each age group except for the general concerns domain. A total of 30 items were initially included under PH; 50 items under MS; 132 items under S; 82 items under P; and 26 items under GC. A response system for the parents was devised which included 0 as *no concern*, 1 as *uncertain,* and 2 as *concerned*. The constructed questionnaire underwent content and construct validation. For the content validation, the appropriateness of the items under each domain and age, and the response system were validated by 3 speech-language pathologists (SLPs) with more than 3 years of experience. The rating was done based on 3 aspects - ‘appropriate’, ‘requires modification’ or ‘can be eliminated’. The content validity index was calculated by dividing the total number of SLPs who rated the item as appropriate/total number of SLPs involved in validation. The items that obtained a content validity score of >0.8 were considered, after which the items were finalized for the data collection. The total number of items in the questionnaire that were included for the final field testing were 28 in PH; 25 in MS; 66 in S; 41 in P; and 13 in GC (Appendix). The construct validation of the questionnaire was ascertained after the completion of the data analysis of the retrieved samples. The results pertaining to the efficacy of the questionnaire have been indicated in the results section, when comparing the typically developing and the CWRELD group.

In phase 2, the parents who participated in the pilot study were not considered for the data collection. Prior to the commencement of the data collection, the participants (mother/father) and their child’s demographic data were collected. The developed questionnaire was administered individually to each of the participants (from both TD and CWRELD groups) from each age group. The questionnaire administration was carried out through an interview method. The questions were asked to each participant who had a child who fit under a specific age group (Group I, II, III, IV, V, or V). All questions were asked for the child’s chronological age level and below, up till 1 year of age. For example, a participant who has a child falling under Group III, were asked questions pertaining to the domains of 4.6 - 5 years of age, and all questions of all age groups (Group I and II) below the target age group. Each participant took 15-20 minutes to complete the questionnaire. It took more duration when the participants responded descriptively to the items as well as when they were doubtful or unaware of the questions asked to them.

In phase 3, the data obtained from the two groups (TD children and CWRELD) were subjected to statistical analysis using SPSS 17 version. Descriptive statistics was done to obtain the mean and standard deviation (SD) for the total scores of each of the domains (PH_Total, MS_Total, S_Total, P_Total, and GC_Total) under each age group for both the TD and CWRELD groups. The results of the descriptive statistics were used to establish a hierarchical representation of parental concerns. Chi square analysis was used to determine the level of significance between parental concerns of TD children and CWRELD groups.

## 3 RESULTS

The results of the descriptive statistics revealed that, the overall concerns exhibited by parents of CWRELD were significantly higher than that of parents of TD children. The mean and SD of concerns exhibited by both parents of TD children and CWRELD are depicted in Fig. (**1**).

The mean and SD of concerns exhibited by parents of CWRELD with/secondary to different disorders across the five domains are represented in Table **2**.

Fig. (**2**) represents the mean and SD of concerns exhibited by both parents of TD children and CWRELD as a function of age (3.7-6.6 years).

The parental concerns that were not mentioned for certain age groups indicated that no concerns were reported. Chi square analysis was used to obtain the level of significance of parental concerns of TD children in comparison to CWRELD across the five domains. Statistically significant findings were obtained across all the domains between TD children and CWRELD (p<0.05). The statistical test values obtained were for Phonology [χ (19) = 59.105, *p<*0.001], Morpho-syntax [χ (21) = 61.199, *p<*0.001], Semantics [χ (34) = 101.00, *p<*0.001], Pragmatics [χ (29) = 85.428, *p<*0.001] and General concerns [χ (15) = 37.600, *p=*0.001].

## 4 DISCUSSION

On considering the mean values of parental concerns, a significant difference was observed across the TD children and CWRELD, with parental concerns being higher in CWRELD compared to TD children. It was observed that minimal parental concerns were reported in the TD group. Here, for the PH domain, items PH15 and PH16 majorly contributed to the concerns that were addressed by the parents. These concerns were pertaining to *the ability of familiar and/or unfamiliar persons to understand the child’s speech.* As observed in the current study, parents portrayed fewer concerns about the understanding of their child’s speech by familiar and unfamiliar persons. As earlier reported, preschool children are usually found to be more intelligible when talking with a variety of communicative partners [21]. Similarly, for the MS domain, item MS24 was majorly addressed as a concern by the parents and it pertained to the *usage of complex sentences*. The subtle concerns exhibited by parents in MS domain can be attributed to the *lack of awareness in young talkers about the commonality among words belonging to the same syntactic categories* (e.g., verb, noun) [22]. Typically developing children tend to have a rich vocabulary, syntax and narrative skills which are determined through decontextualized talk [23]. In the P domain, items P24, P26 and P27 did majorly contribute to the concerns that were addressed by the parents. These concerns were pertaining to the *child’s preference for cartoons, usage of longer dialogues,* and *informal words*. Compared to PH, MS and P, the GC domain achieved the highest mean score, indicating that parents were having greater general concerns about their TD children. These concerns which were GC4, GC11, GC10, GC1, GC2, GC9, GC13, GC6, addressed issues related to the *parent’s time spent for qualitative interaction with the child, sharing ability of child, interest towards books, having number concepts, and interest in extracurricular activities*. In contrast to the parental concerns addressed in PH, MS, P and GC, the S domain did not portray any concerns. These findings were in accordance with the study done by Brown, MacAdam-Crisp, Wang, and Iarocci [24], who indicated higher satisfaction levels in families with children without any disability.

When considering the results obtained by the CWRELD group, the greatest concern was evident in the S domain, indicating that parents were more concerned about the semantic comprehension and usage by their children. All 66 items did contribute to the mean score of 36.49 in the S domain. The highest frequency of concerns was observed to be in S52, S47, S49, S54, S50, S48, S46, S32, S23, S22, S58, S56, S9 that addressed issues related to *naming ability, ‘wh’ questions, usage of objects, knowledge of the semantic category, and understanding word meaning*. Studies done by Goodwin, Fein, and Naigles [25] did indicate that delay in the development of the comprehension of wh-questions could be linked to the child’s overall language level. In addition to this, studies have indicated that the degree of knowledge represented in the child’s semantic lexicon does make words more or less vulnerable to retrieval failure [26] and vocabulary errors were prominent characteristics of developmental disorders [27]. In the P domain, all 41 items did contribute to the mean score of 18.73. The highest frequency of concerns was observed to be in P29, P26, P12, P15, P36, P13, P11, P20, P18, P21, P23, P28, which addressed issues related to *story narration, asking permission, providing descriptive details* and *engaging with other children*. Brinton and Fujiki [28], found children with language disorders to exhibit less interactive skills while using choice questions, requesting for clarification, and responding to elicited speech acts. These concerns may be attributed to the cognitive dysfunctions observed in receptive and expressive language impairments seen in ASD [29], and ADHD [30]. In MS domain, all 25 items did contribute to the mean score of 15.02. The highest frequency of concerns was observed to be in MS20, MS21, MS23, MS24, MS22, MS9, MS25, MS15, MS10, MS19, MS14, MS8, which addressed issues related to the *usage of pronouns, plurals, present and past tense* and *formation of simple sentences*. These concerns exhibited by parents can be attributed to the language that is significantly less complex in individuals with receptive and expressive language impairments seen in ASD [31]. Donahue, Pearl, and Bryan [32] also indicated that the language disturbances observed in children with LD included the use of shorter main clauses on a simple communicative task. In PH domain, all 16 items did contribute to the mean score of 9.39. The highest frequency of concerns was observed to be in PH16, PH14, PH9, PH10 and PH3, which addressed issues related to the *production of speech sounds, pronunciation and the ability of understanding*. These findings were supported by Thomas-stonell, Oddson, Robertson, and Rosenbaum [33] who stated that parents of pre-schoolers with disabilities were more concerned about their child’s difficulties in vocalizing and producing speech sounds for communication needs. Item PH15 (concern regarding familiar person able to understand the child’s speech) did show a less concern compared to PH16 (concern regarding strangers being able to understand the child’s speech). These results were in line with reports that stated that the same pattern of errors becomes more intelligible as the listener becomes familiar with the pattern [34]. Similarly, in GC domain, all 13 items did contribute to the mean score of 5.94. The highest frequency of concerns was observed to be in GC4, GC1, GC11, GC13, GC12, GC6 and GC9 which addressed issues related to *parent’s time spent for qualitative interaction with the child, sharing ability of child, interest towards books, having alphabet knowledge, and having number concepts*. Studies have reported parental concerns of pre-schoolers with disabilities, pertaining to their relationship with peers and their readiness for school [33]. These results do expand the findings by Neece and Baker [35] who stated increased stress levels by mothers of developmentally disabled children than children without any disabilities.

Considering the parental concerns, the results of the descriptive statistics revealed a significant difference between CWRELD with/secondary to different types of disorders. Among the different types of disorders, the highest mean score of parental concerns was reported by the GDD group. These findings were supported by Paquette and Japel [36] who stated that parents were concerned about behaviours, emotions, cognition, communication, socialization, education and health, when there is a delay in one or more developmental areas. The internal sources contributing to the parents level of developmental concerns do include comparison with other children, a perceived level of delay, abnormal child behaviours, and not having the specific knowledge about child development [37]. When considering the parental concerns of children with ASD across the domains in the current study, the S domain did exhibit the highest parental concerns compared to the PH domain, which had the least concerns. The concerns in the domain P and MS were lower than the S domain, but higher than the concerns in the GC domain. Geurts and Embrechts [38] state that parents are more focused on structural language difficulties when children are young, while the focus changes towards pragmatic aspects as they grow older. Beyond the pragmatic impairments, the presence of syntactic deficits was observed in ASD [31]. In the parents of children with ADHD, domain P did elicit the highest parental concerns. Studies have reported evident pragmatic deficits (use of context, use of non-verbal communication, and quality of social relationships) in children with ADHD [38] which is in consensus with the findings of the present study. Considering the mean values, the lowest parental concerns were exhibited by the ID group except in the PH domain. Across the domains in ID, PH domain exhibited the highest parental concerns. These results obtained were in contrast to the findings of Floyd and Olsen [39] who suggested the usage of fewer problem solving skills and less engagement in interactions by pre-schoolers with ID.

Considering the mean values of parental concerns in S domain across the age groups, the 4.1 to 4.6-year-olds obtained the greatest concerns; the 4.7 to 5.0-year-olds obtained the highest concerns in PH domain; while the 6.1 to 6.6-year-olds obtained highest concerns in domains MS, P and GC. The results did not depict any significant pattern of concerns across the age groups. On contrary, a study done by Kaitz [3] suggested that maternal concerns were higher in younger ages compared with later ages. The possible reason for having an inconclusive finding while considering age could be because of the inclusion of the different types of disorders in the CWRELD group. Among them, the initial parental concerns of CWRELD secondary to GDD may be mostly the physical constraints experienced by the child. Once the child does reach a higher age, these parental concerns may tend to focus on communication skills, as these skills may be more demanding due to societal pressure. As the children with GDD fell under the 4.1- 4.6 and 4.7-5.0 year-old groups, there did exist a significant rise in the parental concerns, as it was earlier indicated as well, that parental concerns were higher in this group. Since the older group (6.1- 6.6-year-old) did include parental concerns of children with CWRELD with/secondary to ASD, SSD, ADHD, LD, ID, there was a corresponding increase in the number and variety of concerns as compared to its preceding group (5.7-6.0-year-old).

In the present study, since the numbers of items were not equally distributed across the domains, this would have likely resulted in an overestimation of concerns in certain domains which had the highest number of items that were addressed, compared to domains which had the least number of items to address the concerns. Although utmost homogeneity was tried to be maintained in the CWRELD group, this group did comprise of six heterogeneous disorders either causing or associated with a receptive and expressive language disorder.

## CONCLUSION

The findings of this study do provide insights into the concerns among different types of disorders and also concerns about functions at different ages. It can be inferred that parental concerns did contribute to the development and the well-being of the child. The developed questionnaire can be used in clinical settings which helps to track parental concerns of TD children and CWRELD. The findings from the present study may help pave the way to understand parental concerns which may aid in counselling and in the early identification of the children at risk of various communication disorders. Additionally, this questionnaire may be considered for monitoring parental concerns throughout the course of the intervention program.

## Figures and Tables

**Table:** 

**PHONOLOGY**
**1 - 1.6 YEARS**
Are you concerned that (a) your child is not producing sounds when someone speaks to him/her; (b) your child repeats sounds of others again and again; (c) your child is not able to produce the entire word; (d) a familiar person is unable to understand what your child is saying; (e) a stranger is unable to understand what your child is saying?
**1.6 - 2 YEARS**
Are you concerned that (a) your child uses sounds rather than words; (b) your child repeats only with tone rather than words; (c) your child is not even producing simple words; (d) a familiar person is unable to understand what your child is saying; (e) a stranger is unable to understand what your child is saying.
**2 - 2.6 YEARS**
Are you concerned that (a) your child misses the last part of words while he talks; (b) your child is using one sound for another; (c) a familiar person is unable to understand what your child is saying; (d) a stranger is unable to understand what your child is saying
**2.6 - 3 YEARS**
Are you concerned that (a) your child is not able to say all the sounds; (b) your child is using unclear words most of the time; (c) a familiar person is unable to understand what your child is saying; (d) a stranger is unable to understand what your child is saying
**3 - 3.6 YEARS**
Are you concerned that (a) your child is using the last part of words most of the time; (b) your child is not trying to speak words which go by the order of pronunciation; (c) a familiar person is unable to understand what your child is saying; (d) a stranger is unable to understand what your child is saying
**3.6 - 4 YEARS**
Are you concerned that (a) your child misses some sounds from words; (b) a familiar person is unable to understand what your child is saying; (c) a stranger is unable to understand what your child is saying
**4 - 6 YEARS**
Are you concerned that (a) your child is not able to say the sounds properly all the time; (b) a familiar person is unable to understand what your child is saying; (c) a stranger is unable to understand what your child is saying

**Table:** 

**MORPHO-SYNTAX**
**1 - 1.6 YEARS**
Are you concerned that your child (a) addresses others with names instead of ‘he’, ‘she or ‘it’; (b) is responding only in one or two words
**1.6 - 2 YEARS**
Are you concerned that your child (a) is not using appropriate prepositions in sentences; (b) does not use the word ‘No’ if he/she doesn’t want something; (c) is not able to use words in speech which may indicate a person’s ownership; (d) refers to himself with his name; (e) uses nouns mostly in his utterances
**2 - 2.6 YEARS**
Are you concerned that your child is not (a) using ‘a’ and ‘the’ in sentences; (b) able to use ‘ing’ form with words; (c) using past tense correctly; (d) able to use pronouns correctly; (e) even trying to ask basic questions; (f) able to combine words to form sentences
**2.6 - 3 YEARS**
Are you concerned that your child (a) is not able to form simple sentences with is/am+ing properly; (b) is not able to form sentence with ‘is + adjectives’; (c) is not able to understand the meaning of ‘not’; (d) is not properly using the words ‘here’ and ‘there’ in sentences; (e) is not able to use future tenses in sentences; (f) is not able to understand the ‘est’ concept; (g) has confusion with the usage of he or she
**3 - 4 YEARS**
Are you concerned that your child is not using (a) the present tense properly; (b) plural forms correctly; (c) ‘is’, ‘are’, ‘am’ in sentence properly
**4 - 6 YEARS**
Are you concerned that your chid is not using (a) ‘if’ and ‘so’ words while forming the sentence; (b) words like ‘our’, ‘they’, and ‘their’
**SEMANTICS**
**1 - 1.6 YEARS**
Are you concerned that your child (a) understands only simple commands; (b) does not use gestures or pointing for the things that he wants; (c) points only to familiar objects; (d) does not state certain objects to be his; (e) is not able to understand and point out to body parts; (f) is not able to identify specific objects from a group; (g) is not becoming aware of others emotions; (h) uses gestures while producing sounds; (i) uses fewer words; (j) is not answering for simple questions like ‘what is this’; (k) does not ask for more things when he/she wants
**1.6 – 2 YEARS**
Are you concerned that your child (a) doesn’t understand most of the words spoken; (b) does not listen to, when pictures are being named; (c) doesn’t show interest in listening to the stories; (d) is not responding appropriately for yes/no questions at least with a headshake; (e) does not understand that the object are there, if they are not visible or hidden; (f) doesn’t know to categorize food from others; (g) imitates only the present events and not the past; (h) is not using names of familiar objects; (i) is not able to make animal sounds and name the animals; (j) is not able to convey his/her toilet needs; (k) is unable to identify and name pictures; (l) is not saying his name when asked to; (m) is not responding immediately
**2 - 2.6 YEARS**
Are you concerned that your child (a) does not comprehend most of the words; (b) is not able to listen to the stories for few minutes?; (c) is unable to follow a series of related commands; (d) is unable to identify pictures with action; (e) does not know the difference between one and many; (f) does not know the use of objects; (g) is unable to repeat the numbers correctly; (h) is not answering to the questions that were asked to him
**2.6 - 3 YEARS**
Are you concerned that your child (a) is not able to point to pictures when they are described by their use; (b) does not know the ‘boy’ and ‘girl’ concepts; (c) is not able to understand the ‘in’ and ‘out’ concepts; (d) does not know ‘big’ and ‘small’ concepts; (e) is not able to match the things; (f) is not responding to ‘yes’ or ‘no’ questions; (g) is unable to answer, questions like ‘who’, ‘why’, ‘where’, when’, ‘how many’; (h) is not responding correctly to the situation specific questions
**3 - 3.6 YEARS**
Are you concerned that your child is not able to (a) comprehend and use ‘in front of’ and ‘behind’ properly; (b) to identify hard and soft, rough and smooth; (c) identify shapes; (d) respond to commands; (e) answer simple questions; (f) say reasons for the questions asked; (g) ask too many ‘what’ and ‘who’ questions; (h) count the objects by pointing
**3.6 - 4 YEARS**
Are you concerned that your child is (a) not able to answers to ‘how much’ and ‘how long’ questions; (b) not able to say the events in order of its sequence properly; (c) mixing real and unreal events in the story; (d) not engaging in long detailed conversations with others; (e) not able to answer ‘what if’ questions
**4 - 4.6 YEARS**
Are you concerned that your child is not able to (a) comprehend ‘between’, ‘above’, ‘below’, ‘top’ and ‘bottom’ correctly; (b) repeat digits
**4.6 - 5 YEARS**
Are you concerned that your child is not able to (a) answer complex questions; (b) identify an object with its form, colour and use; (b) ask word meanings; (c) repeat days of weeks in correct sequence
**5 - 6 YEARS**
Are you concerned that your child (a) does not understand opposites; (b) is not able to know difference between AM and PM; (c) does not know the difference between a coin and a rupee; (c) is not able to comprehend half and whole; (d) is not able to describe the ‘location’ words; (e) is not able to understand the positions like first, second and third; (f) is not able to say days of a week in order

**Table:** 

**PRAGMATICS**
**1 - 1.6 YEARS**
Are you concerned that your child does (a) not bring things to show others; (b) not ask for the things that he/she wants; (c) not try to get others attention by sounds, touching or by calling the other person; (d) not say ‘hi’, ‘bye’, and ‘thank you’; (e) not say ‘no’ or a gesture if he doesn’t want something; (f) not try to say about objects to grab another person’s attention; (g) answer in simple form which is not well understood by everyone; (h) not respond by looking at you or repeating what was said; (i) make gestures along with sounds in order to tease and scold; (j) not imitate
**1.6 - 2 YEARS**
Are you concerned that your child does not (a) use words or phrases to express the needs; (b) name objects that he knows in front of others; (c) draw attention by asking ‘what is that’; (d) use simple words to express problems or even saying that something belongs to him; (e) wait for his/her turn to speak; (f) talk to self while playing; (g) do things independently
**2 - 3 YEARS**
Are you concerned that your child is (a) not interested in engaging in short dialogues with others; (b) not able to verbally introduce and change topics of discussion; (c) not using long imaginative ways to describe something; (d) not providing descriptive details for the understanding of others; (e) not asking for any clarifications; (f) not interested in other children while playing; (g) more involved in cartoons than talking or playing with others; (h) not using objects, actions or ideas to denote other things while playing
**3 - 4 YEARS**
Are you concerned that your child is not (a) able to use longer dialogues; (b) able to use words like ‘uh-huh’, ‘yeah’, ‘ok’ while others are talking; (c) talking in a different way to very young children; (d) able to ask for permissions; (e) using language for jokes and fantasies; (f) able to put the sentence in different way when the listener has not understood; (g) able to correct others; (h) able to play with a group of children; (i) able to imagine the role of another person in play
**4 - 5 YEARS**
Are you concerned that your child is (a) not able to express feelings and emotions; (b) not able to tell long stories; (c) not able to identify missing parts in pictures; (d) much of self praises; (e) having no interest in completing what he/she starts
**5 - 6 YEARS**
Are you concerned that your chid (a) will give you fear and insult you in front of others; (b) is not able to play any games with rules

**Table:** 

**GENERAL CONCERNS**
Are you concerned that (a) your child is not careful about the dangers; (b) your child is too shy or self-centred; (c) your child is always prefers to be alone than being with other children; (d) you are not spending enough time to talk and play with the child; (e) your child is not able to talk because of the overuse of gadgets; (f) your chid is not talking or playing because he/she is a single child; (g) your chid is not responding because you are not friendly with the child; (h) your child is not talking because you are not talking at child’s level; (i) your child is not sharing anything with others; (j) your child is not interested in any extracurricular activities like singing, drawing, dancing, etc.; (k) your child does not show any interest in books; (l) your child hasn’t yet started identifying alphabets; (m) your child is not able to understand the number concept

**Fig. (1):**
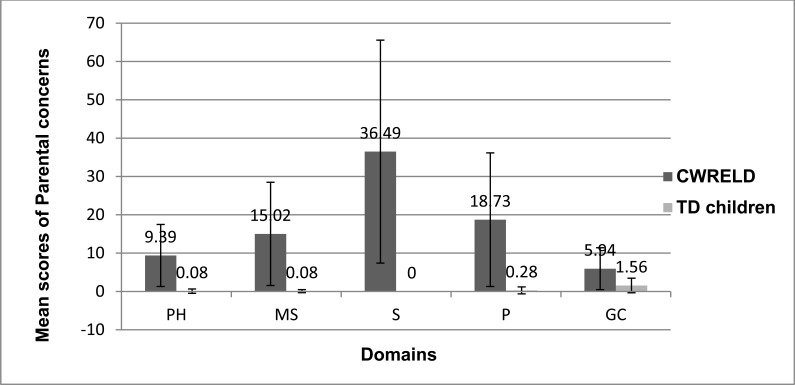
Comparison between parental concerns of TD children and CWRELD. Note: PH-Phonology; MS-Morpho-syntax; S-Semantics; P-Pragmatics; GC- General Concerns.

**Fig. (2):**
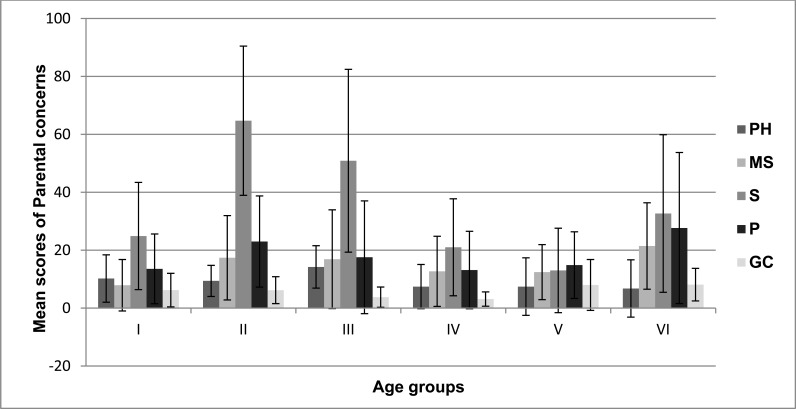
Parental concerns of CWRELD as a function of age across the domains. Note: PH-Phonology; MS-Morpho-syntax; S-Semantics; P-Pragmatics; GC- General Concerns

**Table 1:** Total number of TD children and CWRELD under each age group.

**S. No**	**Groups**	**Age Group** **(in years)**	**TD** **children**	**CWRELD**
1	I	3.7-4.0	9	9
2	II	4.1-4.6	8	10
3	III	4.7-5.0	8	9
4	IV	5.1-5.6	9	7
5	V	5.7-6.0	8	7
6	VI	6.1-6.6	9	9

**Table 2:** The total number of disorder specific samples and the Mean±SD of parental concerns of CWRELD with/secondary to different disorders.

-	**Type of Disorder**	**Total No. of Samples**	**Domains (Mean±SD)**				
			**PH**	**MS**	**S**	**P**	**GC**
-	**CWRELD**	37	9.81±8.14	15.78±13.54	35.30±27.09	16.97±16.47	5.65±5.83
**CWRELD** **with/** **secondary** **to**	**ASD**	5	3.60±2.19	13.20±8.11	40.60±21.86	28.40±11.99	7±2.24
	**SSD**	3	10±5.29	3.67±6.35	22±15.72	6.33±7.10	3.67±4.73
	**ADHD**	1	7	26	41	55	10
	**LD**	2	0	15.50±14.85	11±4.24	8±7.07	6.50±3.54
	**ID**	1	14	0	9	4	0
	**GDD**	2	23.50±6.36	24±31.11	107±25.46	45.50±21.92	12.50±4.95

Note: ASD-Autism Spectrum Disorder; SSD-Speech Sound Disorder; ADHD-Attention Deficit Hyperactivity Disorder; LD-Learning Disability; ID; Intellectual Disability; GDD- Global Developmental Delay.

## Data Availability

The data source of the current study cannot be revealed as it contains sensitive information on the patient’s disability profile.

## APPENDIX

### A Parental Questionnare To Determine The Communication Profile Of Typically Developing Children And Cwreld

This questionnaire is developed to profile the communication concerns of parents of children with receptive and expressive language disorders. This questionnaire contains age specific items categorized under 5 domains (phonology, morpho-syntax, pragmatics, semantics and general concerns). The targeted age groups are children between 1 and 6 years of age.

Each sub question under each component and age range to be rated: 0 (No concerns), 1 (Uncertain), 2 (Concerned).
